# Gender diversity in pediatric surgery: academic ranks and scholarly productivity amongst pediatric surgeons

**DOI:** 10.3389/fsurg.2024.1442501

**Published:** 2024-08-01

**Authors:** Danielle M. Mullis, Claudia Mueller, Spencer A. Bonham, Emily Hunt, Daniela Uribe, Hayley Miller, Y. Katherine Bianco

**Affiliations:** School of Medicine, Stanford University, Palo Alto, CA, United States

**Keywords:** gender, disparities, pediatric surgery, pediatric, surgery

## Abstract

**Purpose:**

Despite a growing number of women entering medical school, a small proportion of women pursue surgical specialties, including pediatric surgery. This multi-center study assesses gender-based differences in measures of scholarly productivity and distribution of faculty positions.

**Methods:**

This is a retrospective web-based study of all pediatric surgeons at twelve large institutions across the United States. Data published by the American Association of Medical Colleges was compiled and analyzed to understand the gender distributions of medical students, general surgery residents, and pediatric surgery fellows. *P*-values were calculated using two-sided Student's independent t-tests and chi-squared tests.

**Results:**

There have been a growing number of women applying into pediatric surgery, but the proportion of women matriculating into these fellowships is not concordant. Women are still underrepresented (28%) amongst the pediatric surgeon workforce. A total of 111 pediatric surgeons were identified for this study, which included 31 women (28%) and 80 males (72%). There was a significant difference in the distribution across academic ranks between genders (*p* < 0.001). Women had significantly fewer publications per year after residency, fewer total publications, and a lower h-index in comparison to men (*p* < .001, *p* = .005, *p* = .002, respectively).

**Conclusions:**

Women are not only underrepresented in pediatric surgery, but there are also significant differences in the distribution of faculty positions and scholarly productivity when comparing men and women. There is a pressing need to improve gender diversity and identify barriers that may prevent women from advancing to leadership positions and achieving professional success.

## Introduction

Women historically have been underrepresented in medicine (1, 2). However, there has been a recent surge in the number of women entering medicine, and as of 2017, the number of women matriculating to medical school surpassed the number of men (3). This trend reflects our society's movement towards gender equality and recognition of the valuable contributions that women make in historically male-dominated professions. However, it is well-known that there is still much work to be done in terms of increasing women's representation in academic medicine; according to the AAMC's latest report “The State of Women in Academic Medicine: Exploring Pathways to Equity,” women hold 41% of full-time faculty positions and 18% of all department chair positions (4). It is well-known that there is a significant gender disparity across almost all surgical subspecialities (5, 6).

Pediatric surgery is one of the many surgical specialties which continues to be dominated by male surgeons (7). As of 2023, only 28% of all pediatric surgeons in the U.S. were women (8). The path to becoming a pediatric surgeon is very long; after completing medical school, aspiring pediatric surgeons must first complete a general surgical residency (9). Various studies have reported that women experience gender-based challenges during surgical residency (6, 10–12). After general surgery residency, aspiring pediatric surgeons must then complete a pediatric surgery fellowship (9). There has been relatively little research done on gender disparities in pediatric surgery fellowships and amongst practicing pediatric surgeons. One recent article found that significant gender differences exist in letters of recommendations written for women applying to pediatric surgery fellowship positions (13). Alternatively, another study identified that women's representation at pediatric surgery conferences has significantly improved, with half of all participants identifying as women (14).

Prior research has identified many metrics that define the success of physicians in their fields including research productivity, salary, and leadership (15, 16). Women in academic medicine, despite having similar aspirations and the same dedication to their work as their male counterparts are often overlooked for leadership roles (15). These trends also have been found amongst surgical specialties, as research overwhelmingly shows that women in surgery have lower research productivity, are paid less, and have less leadership roles than their male counterparts (16–18). Academic success has been previously described by numerical information including Hirsch's index (h-index), number of publications, and grant funding (19–22). Studies have shown that male surgeons are more likely to hold leadership roles, have higher academic ranks and higher salaries than female surgeons (16, 23, 24).

Given that women are in the minority of faculty positions within pediatric surgery, this study aims to assess the prevalence and nature of gender-based differences within the field of pediatric surgery at twelve large institutions across the northern, southern, eastern, and western United States. This study collected data and performed detailed analysis on just over 10% of all pediatric surgeons practicing in the U.S. We assessed gender-based differences in academic rank and achievement including number of publications (both during and after residency), number of advanced degrees obtained, and h-indexes. We hypothesized that males in this cohort would have higher academic rankings and higher values for measures of academic productivity including h-index scores, number of publications, with a higher proportion of males having advanced degrees.

## Methods

Institutional Review Board approval (protocol 66392) was obtained prior to the start of this study. Twelve public institutions across four different geographical regions of the United States (Northeast, Midwest, South, and West) were selected. Three large academic institutions in each region were selected for further analysis. The names of all faculty members, for the department of pediatric surgery were obtained and analyzed. Emeritus faculty members were excluded from this study. Doximity, a networking platform for medical professionals, Elsevier's Scopus, PubMed, and the twelve institutions' websites were used to gather variables of interest.

Gender was determined by assessing the pronouns used to describe each faculty member. Faculty members were grouped into the following categories: men (referred to with pronouns “him” or “his”), women (referred to with pronouns “her” or “hers”), or other (if pronouns other than “him,” “his,” “her,” “hers”) were used. Elsevier's Scopus was used to gather the h-index for each faculty member. PubMed was used to determine each faculty member's total number of publications. Using each institution's website and Doximity, the institutions of each faculty member's medical school and residency program were recorded. We then recorded whether each faculty member went to a “top-20 NIH-funded” institution; the names of the twenty highest funded institutions in 2022 were obtained from the National Institute of Health's (NIH) Research Portfolio Online Reporting Tools website (25). This tool was also used to identify whether each faculty member had ever received (1) NIH funding of any type and (2) NIH Research Project Grant (R01). To understand trends in terms of gender diversity within the medical field, historic data from the Association of American Medical Colleges (AAMC) was compiled. This included the AAMC's Report on Residents (26), ERAS® Statistics (27), and AAMC's FACTS Report (28).

Statistical analysis was completed using JMP software. Categorical variables were analyzed using Chi-squared tests and continuous variables were analyzed using two-tailed Student's t-tests and linear regression. Averages are reported with standard deviations. Error bars are standard deviations. A *p*-value of less than 0.05 was considered statistically significant.

## Results

**Figure 1:**
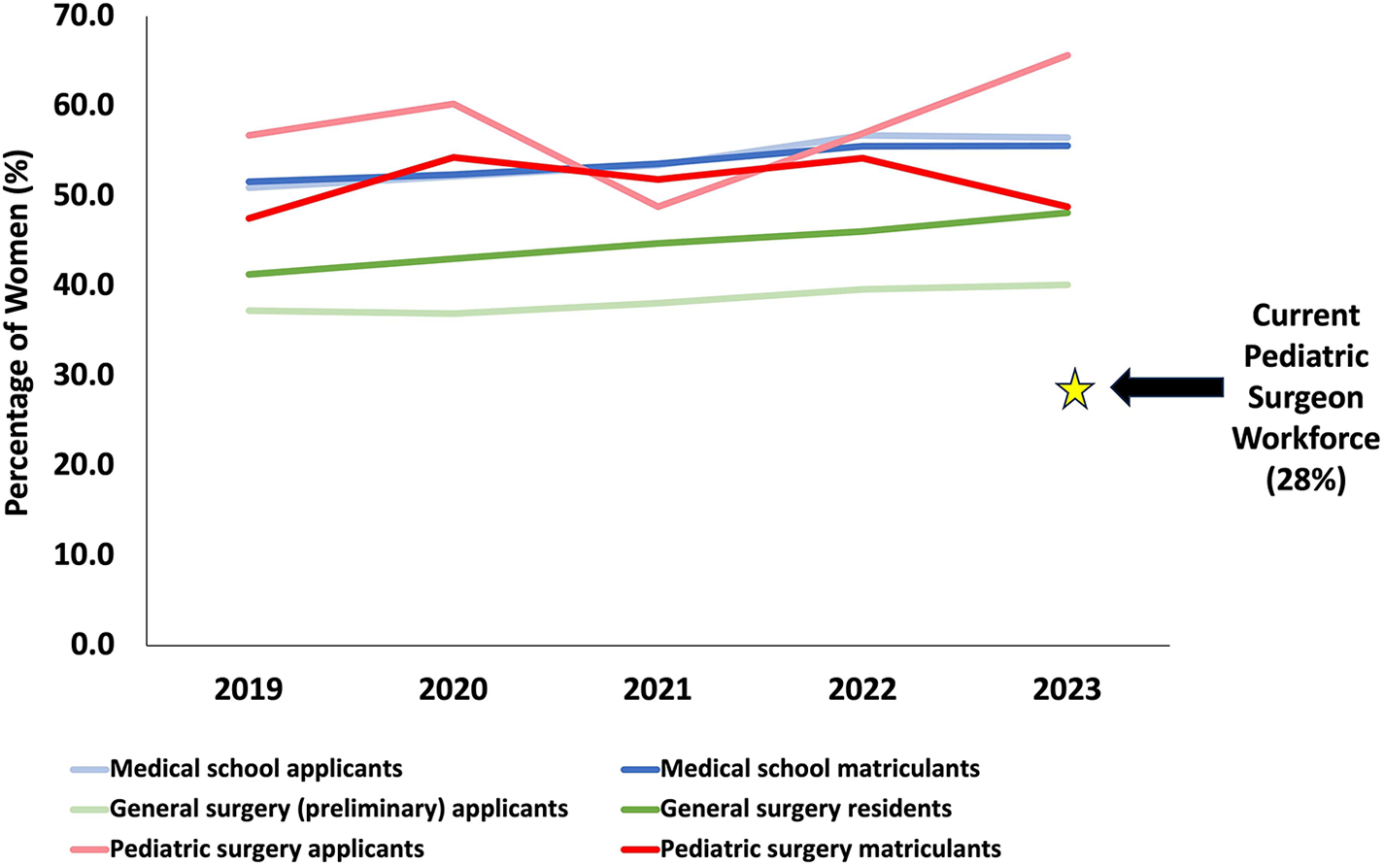
Percentage of women over time in the pediatric surgeon training pathway. This data was compiled from reports published by the American Association of Medical Colleges (8, 26–28).

Data obtained from the AAMC identified that there has been an increasing number of women applying to and matriculating into medical school from 2019 to 2023 (28). In 2019, approximately half of the medial school applicants and matriculants were women (50.9% and 51.6%, respectively); by 2023, the percentages of applicants and matriculants identifying as women had increased to 56.5% and 55.6%, respectively (Supplementary Table S1, Figure 1). The percentage of female preliminary general surgery applicants has been increasing over time, trending from 37.3% in 2019 to 40.2% in 2023 (Supplementary Table S1). The number of female general surgery residents increased from 41.3% in 2019 to 48.2% in 2023 (Supplementary Table S1). Despite the relatively low proportion of female general surgery residents, female pediatric surgery applicants have fluctuated between 48.8% and 65.5% from 2019 to 2023 (Supplementary Table S1, Figure 1). Female matriculants to pediatric surgery fellowship positions remained approximately 50% over the past few years (47.6% in 2019, 51.9% in 2021, 48.8% in 2023) (Supplementary Table S1, Figure 1). The current pediatric surgeon workforce is currently (as of late 2023) made up of 28% women (Supplementary Figure S1).

**Figure 2:**
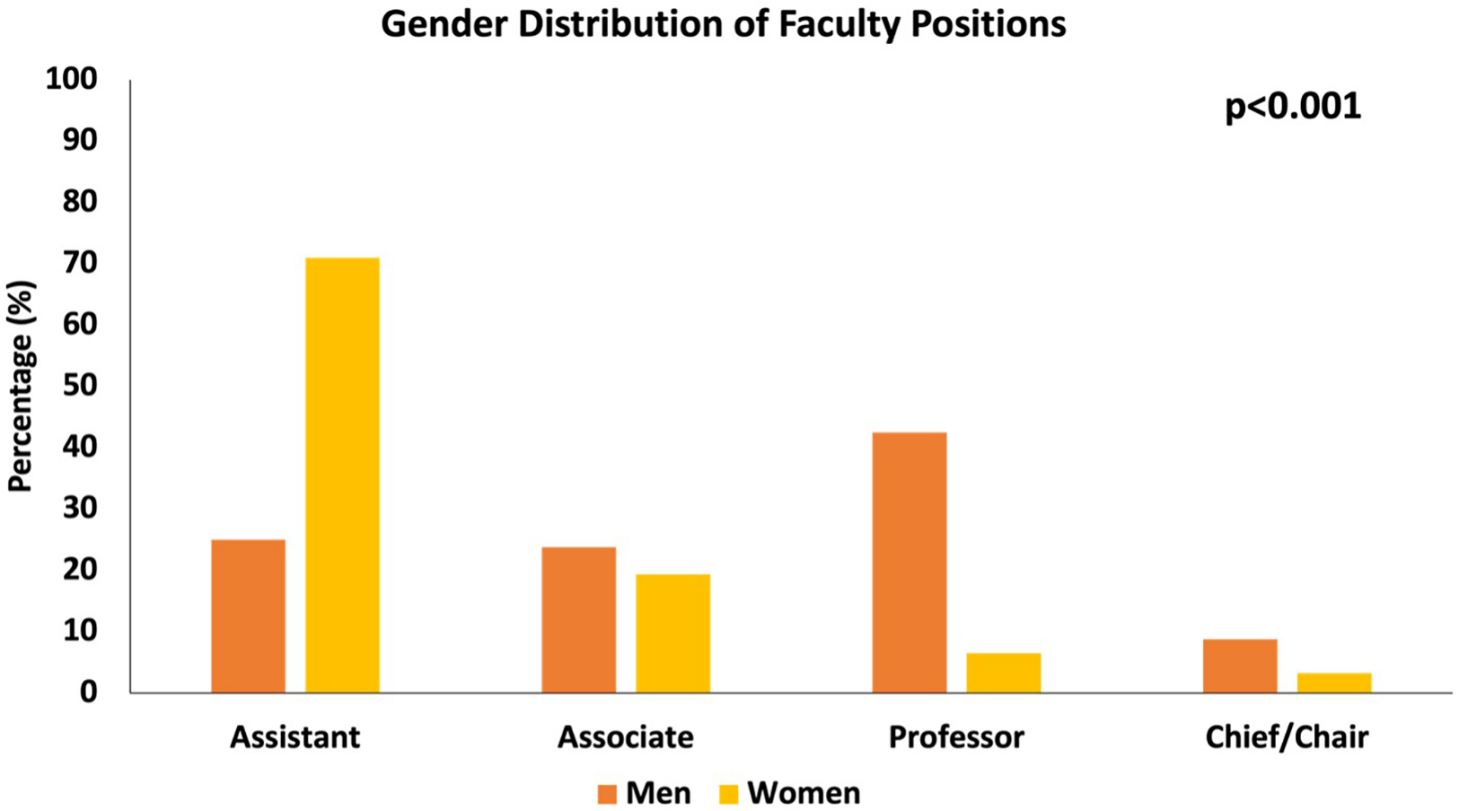
Gender distribution across academic ranks. Data on each person's academic rank at each institution for all 111 pediatric surgeons was collected. Chi-squared analysis revealed there was a significant difference in the distribution across academic ranks (*p* < 0.001).

In late 2023, we identified 111 faculty members across twelve different institutions that were eligible for the study (Supplementary Figure S2, Table S2). Thirty-one were women (28%), eighty were male (72%), and no one was referred to using pronouns other than “she/her/hers” or “he/him/his.” There was a significant difference in the distribution of men and women across academic ranks (*p* < 0.001, Table 1, Figure 2). Sixty-seven percent (20 out of 31) of the women occupied assistant professorship positions, while only 25.0% (20 out of 80) of men occupied assistant professorship positions (Table 1). Only 6.5% (2 out of 31) of women occupied professorship positions, while 42.5% (34 out of 80) of men occupied professorship positions (Table 1). To adjust for lead time bias, a subset of the data was analyzed. The greatest number of years elapsed from residency graduation was 30 years for women; therefore, any men who were more than 30 years out from residency graduation were excluded this sub-analysis (*n* = 12 of 80). When comparing these two cohorts, there was still a significant difference in the distribution of men and women across academic ranks (*p* < 0.001, Supplementary Table S3).

**Table 1:** Gender distribution across academic ranks.

	Men	Women
Assistant Professor	25.0 (20)	66.7 (22)
Associate Professor	23.8 (19)	19.3 (6)
Professor	42.5 (34)	6.5 (2)
Chief/Chair	8.8 (7)	3.2 (1)
Total	100 (80)	100 (31)

Data on each person's academic rank at each institution was collected. Data is presented in the following format: percentage (number). Chi-squared analysis demonstrated there was a significant difference in the distribution across academic ranks (*p* < 0.001).

Next, academic data was analyzed. A similar distribution of men and women attended top-twenty NIH funded medical school and residency programs (*p* = 0.38 and *p* = 0.75, respectively, Table 2). We found there was no significant difference between the proportion of men and women who had obtained advanced degrees (in additional to their medical degrees) in comparison to men (*p* = 0.76, Table 2). There was also no significant difference between the proportion of men and women who completed additional non-research fellowships (*p* = 0.85, Table 2).

**Table 2:** Academic data on pediatric surgeon faculty members.

	Men	Women	*p*-value
Attended a top-twenty NIH funded medical school	27.5 (22)	19.4 (6)	0.38
Attended a top-twenty NIH funded residency	38.8 (31)	35.5 (11)	0.75
Additional advanced degree	20.0 (16)	22.6 (7)	0.76
Completed additional non-research fellowships	11.3 (9)	9.7 (3)	0.85
Received NIH funding	16.1 (5)	16.1 (5)	0.19
Received R01 NIH funding	3.2 (1)	3.2 (1)	0.14

If an institution was one of the twenty that received the most NIH funding in 2022, it was classified as a “top-twenty NIH funded” institution. *P*-values were determined using chi-squared tests.

**Figure 3:**
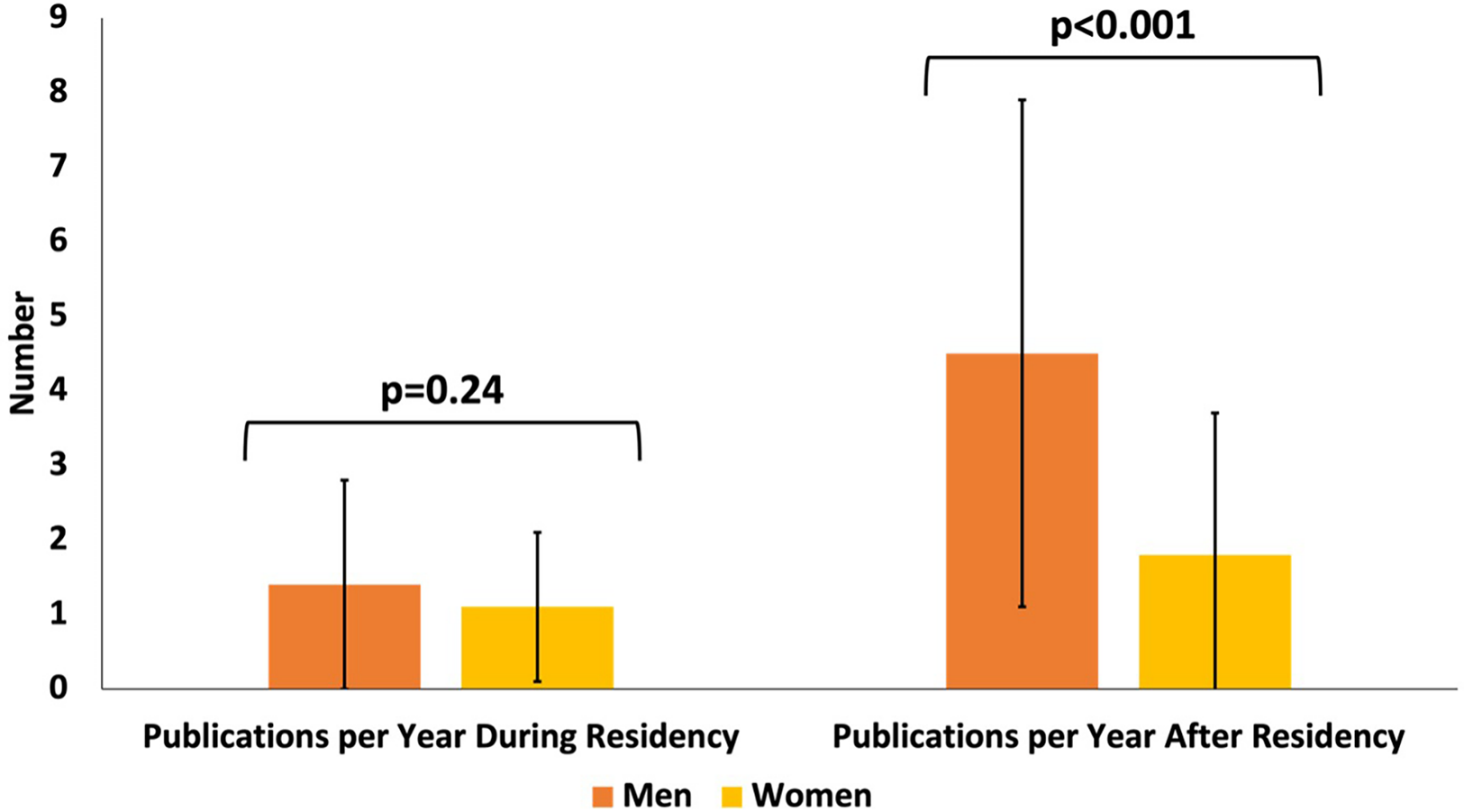
Publication trend for pediatric surgeons by gender. For all 111 pediatric surgeons, total number of publications per year during residency was obtained as well as number of publications per year after residency. There was no difference in the number of publications per year during residency for women compared to men (*p* = 0.24), but there was a significant difference in the number of publications per year after residency (*p* = 0.01). *P*-values were calculated using a two-sided Student's t-test.

Lastly, metrics of academic productivity were analyzed. There was no significant difference between the number of publications during residency nor the number of publications per year of residency for men in comparison to women (*p* = 0.15 and *p* = 0.24, respectively, Table 3, Supplementary Figure S2). Additionally, there was no significant difference between the proportion of men and women who received NIH funding of any type nor an NIH R01 grant (*p* = 0.19 and *p* = 0.14, respectively, Table 2). However, after residency, women published significantly fewer journal articles in comparison to men. Women had significantly fewer total number of publications, publications after residency, publications per year after residency (*p* = 0.005, *p* = 0.01, *p* < 0.001, Table 3, Figure 3, Supplementary Figure S3).

**Table 3:** Measures of academic productivity for pediatric surgeons.

	Men	Women	*p*-value
Publications before residency	0.5 ± 1.8	1.5 ± 4.0	0.09
Publications during residency	9.9 ± 9.9	7.0 ± 6.1	0.15
Publications per year during residency	1.4 ± 1.4	1.1 ± 1.0	0.24
Publications after residency	73.7 ± 86.7	28.7 ± 41.5	0.01*
Publications per year after residency	4.5 ± 3.4	1.8 ± 1.9	<0.001*
Total publications	84.1 ± 88.1	37.3 ± 40.9	0.005*
H-index	24.4 ± 18.8	12.9 ± 10.5	0.002*

Data is presented as the following: average ± standard deviation. *P*-values were determined using two-sided T-tests.

* *p* < 0.05.

Women also had significantly lower h-indexes on average in comparison to men (12.9 ± 10.5 vs. 24.4 ± 18.8, *p* = 0.002, respectively) (Table 3, Supplementary Figure S4). Linear regression demonstrated that for each year elapsed since residency, h-index increases to a greater extent for men than for women (Supplementary Figure S5).

## Discussion

This study collected data on approximately 10% (111 out of 1,075) of the current pediatric surgeon workforce (8). Despite an overall increase in the proportion of women entering medical school, women applying to general surgery residency and matriculating into general surgery residency are still underrepresented. Additionally, while there is generally a high proportion of women applying to pediatric surgery fellowships, a smaller proportion of women have been accepted to these fellowship programs (Supplementary Table S1). Although more women are applying to pediatric surgery programs over the past few years (48.8%–65.7%), the percentage of women who matriculate has been incongruent (47.6%–54.%) (Supplementary Table S1). There is clearly much work to be done to achieve representation in the pediatric surgeon workforce, currently comprised of only 28% women (8) (Figure 1, Supplementary Table S1).

For the 111 pediatric surgeons in our study, there was a significant difference between men and women across academic ranks, with a relatively higher proportion of women occupying assistant professorship positions and a higher proportion of men occupying full professorship and chief/chair positions. These findings are consistent with prior research, which has shown that there is a stark difference between the proportion of women in the field of pediatric surgery and the proportion of women who occupy leadership positions (29). The lack of parity in leadership position indicates that more effort needs to be taken to ensure female physicians in the field are afforded the same opportunities for leadership that male physicians are given.

Analysis of other metrics of academic productivity showed that males in this study published significantly more scholarly work after residency and had a significantly higher h-index when compared to women. Analysis of academic productivity prior to completing residency, such as publications during residency, per year of residency, and number of advanced degrees were not significantly different between men and women. This indicates that gender disparities manifest themselves most prominently after residency, which could be attributed to publication bias, women bearing a disproportionate responsibility of family responsibilities, limited mentorship, limited networking, or inadequate support for research funding (30–34). It is possible that the discrepancies in number of publications is due to lack of opportunities for advancement and support in pursuing leadership positions, which require a certain amount of scholarly productivity. These opportunities may be more equitably given during medical school and residency, where established programming may assist in creating time for the pursuit of research goals.

Diversity is critical to enhancing creativity and fostering physician satisfaction, which both improve the patient experience. The lack of parity across the different levels of leadership in pediatric surgery fosters an environment in which gender discrimination can and does occur (35, 36), leading to a lack of diverse perspectives and innovation within the field. Furthermore, Pediatrics requires extensive amounts of communication with the family and the patient, which is best served when the care team functions harmoniously. A lack of diversity fails to promote teamwork which is crucial for a high-quality patient experience (37).

A concerted effort will be required to address gender disparities amongst pediatric surgeons. More research is needed to understand where the field currently stands. Why are women publishing just as much during residency but then publish less per year than their male counterparts? Is the distribution of academic positions only evidence that most female pediatric surgeons are early in their career, or are there barriers in place that aren't enabling women to be promoted to a full professorship? It is evident that the field is moving in the right direction, but there are clear differences between men and women in the field of pediatric surgery that can be addressed. Implementing supportive, more flexible policies that provide support to the diverse needs of women may further encourage academic productivity (38). Additionally, a department's commitment to addressing bias and discrimination through programs such as unconscious bias training could further raise awareness of gender disparities within the field of pediatric surgery (39).

There is a very important limitation to this study that must be addressed. There were no pediatric surgeons identified for this study who did not have “she/her” or “he/him” pronouns. Therefore, this study only assesses differences between pediatric surgeons we identified as “men” and “women.” However, the use of certain pronouns doesn't always match one's gender identity. Gender is a fluid construct, and more research is needed to assess the representation of all different genders in pediatric surgery to ensure there is adequate representation and equality for people belonging to gender minorities. More data is needed and would enable us to further our understanding of how we can help promote inclusion within academic medicine.

This study is a large, contemporary study of pediatric surgeons at twelve institutions geographically distributed across the United States. While other studies have identified gender disparities in academic medicine (20–22, 40–42) and specifically amongst surgeons (23, 24, 30, 36, 39, 43) and pediatricians (44–46), gender disparities amongst pediatric surgeons remains relatively unexplored. We hope to add to the growing body of literature that seeks to identify gender disparities and understand progress that has been made so we can continue creating a more equitable and inclusive profession.

In conclusion, we have performed a rigorous analysis of approximately 10% of the current pediatric surgeon workforce. We have identified current trends in medical education (residency and pediatric fellowship) that suggest more could be done to increase the number of women in pediatric surgeon along several stages of the training pathway. Additionally, we highlight some gender disparities that exist and propose some possible explanations for these observations. Future work should include interviews and focus groups to provide deeper insights into the factors that (1) deter women along the pediatric surgeon training pathway and (2) prevent women from publishing as much as men.

## Data Availability

The raw data supporting the conclusions of this article will be made available by the authors, without undue reservation.
