# Supplementary material for: Gender diversity in pediatric surgery: academic ranks and scholarly productivity amongst pediatric surgeons
Source: Front Surg. 2024 Aug 1;11:1442501. doi: 10.3389/fsurg.2024.1442501 (PMC11324546; doi:10.3389/fsurg.2024.1442501)
Supplement: Supplementary file 1 [file Datasheet1.docx]

|  | **2019** | **2020** | **2021** | **2022** | **2023** |
| --- | --- | --- | --- | --- | --- |
| **Medical School Applicants** | 50.9 | 52.2 | 53.4 | 56.8 | 56.5 |
| **Medical School Matriculants** | 51.6 | 52.4 | 53.6 | 55.5 | 55.6 |
| **General Surgery (Preliminary) Applicants** | 37.3 | 36.9 | 38.1 | 39.6 | 40.2 |
| **General Surgery Residents** | 41.3 | 43.1 | 44.8 | 46.1 | 48.1 |
| **Pediatric Surgery Applicants** | 56.8 | 60.3 | 48.8 | 57.0 | 65.7 |
| **Pediatric Surgery Matriculants** | 47.6 | 54.3 | 51.9 | 54.2 | 48.8 |
| **Current Workforce – Pediatric Surgeons** |  |  |  |  | 28.0 |

**Supplemental Table 1: Percentage of Women Over Time in the Pediatric Surgeon Training Pathway.** This data was compiled from reports published by the American Association of Medical Colleges [8] [27-29].

|  | **Men** | **Women** | **Total** |
| --- | --- | --- | --- |
| **Northeast** | 18 | 6 | 24 |
| **Midwest** | 27 | 14 | 41 |
| **West** | 16 | 4 | 20 |
| **South** | 19 | 7 | 26 |
| **Total** | 80 | 31 | 111 |

**Supplemental Table 2: Pediatric Surgeon Faculty by Region.** Our study included approximately 10% of the pediatric surgeon workforce. Data was collected on all pediatric surgeons (excluding emeritus faculty) at twelve different institutions across four different geographical regions (three institutions per each geographic region).

|  | **Men** | **Women** |
| --- | --- | --- |
| **Assistant Professor** | 29.4 (20) | 66.7 (22) |
| **Associate Professor** | 27.9 (19) | 19.3 (6) |
| **Professor** | 36.8 (25) | 6.5 (2) |
| **Chief/Chair** | 5.9 (4) | 3.2 (1) |
| **Total** | 100 (68) | 100 (31) |

**Supplemental Table 3: Gender Distribution Across Academic Ranks for Younger Faculty.** A subset of the data was analyzed to adjust for lead time bias. The greatest number of years elapsed from residency graduation was 30 years for women; therefore, any men who were more than 30 years out from residency graduation were excluded this sub-analysis (n=12 of 80). Data is presented in the following format: percentage (number). The Chi-squared analysis demonstrated there was a significant difference in the distribution across academic ranks (**p<0.001**).

**Supplementary Data Figure 1**

**
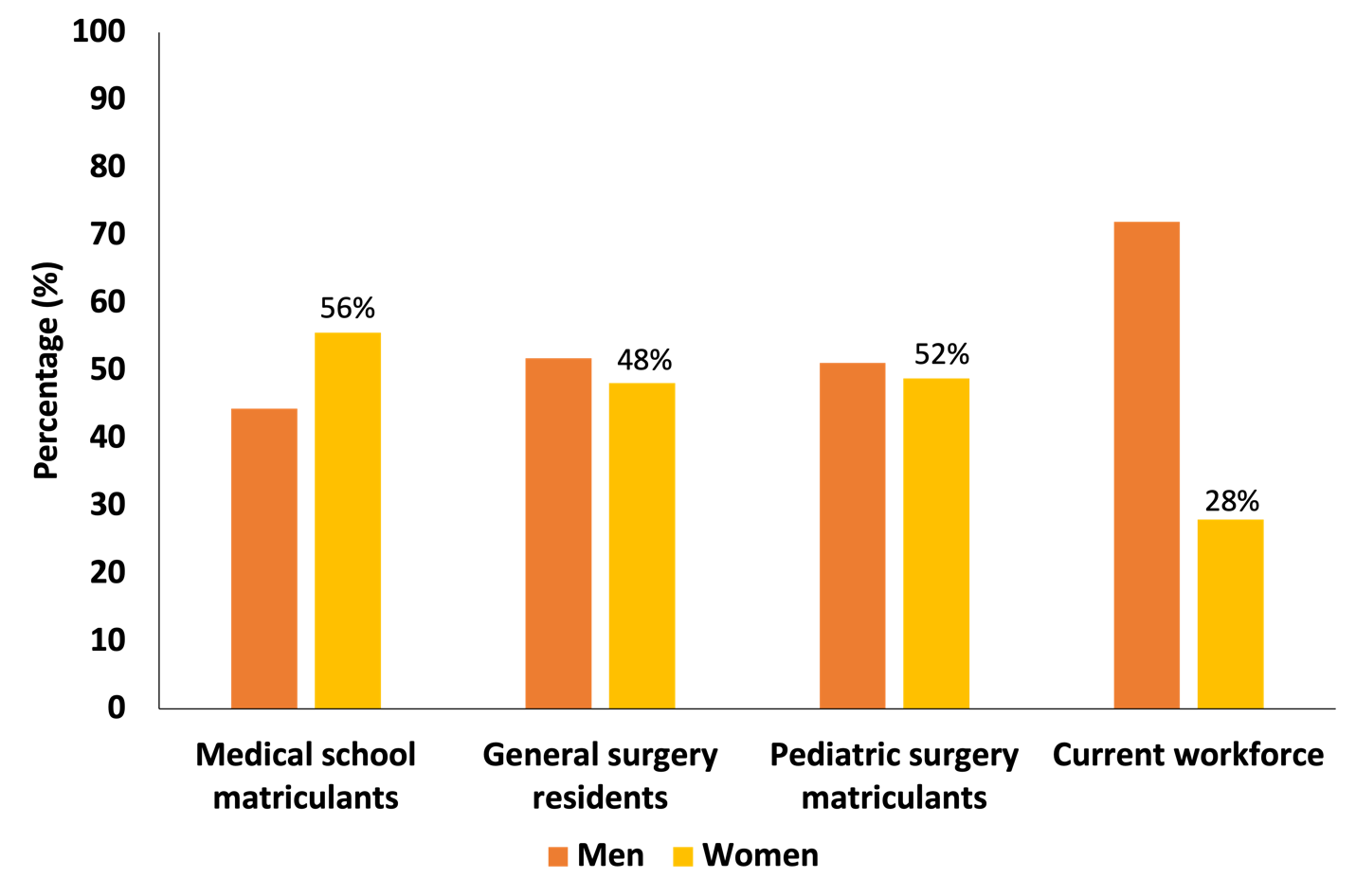
**

**Supplementary Data Figure 2**


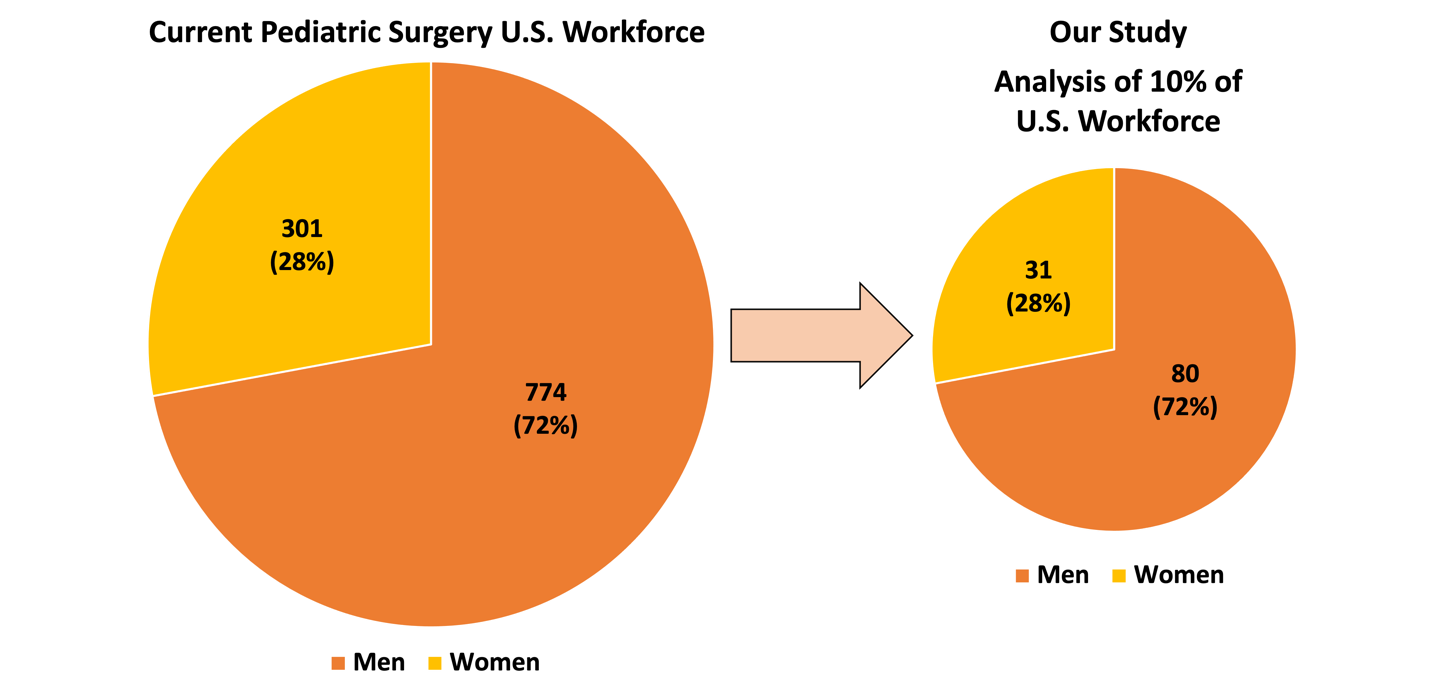


**Supplementary Data Figure 3**


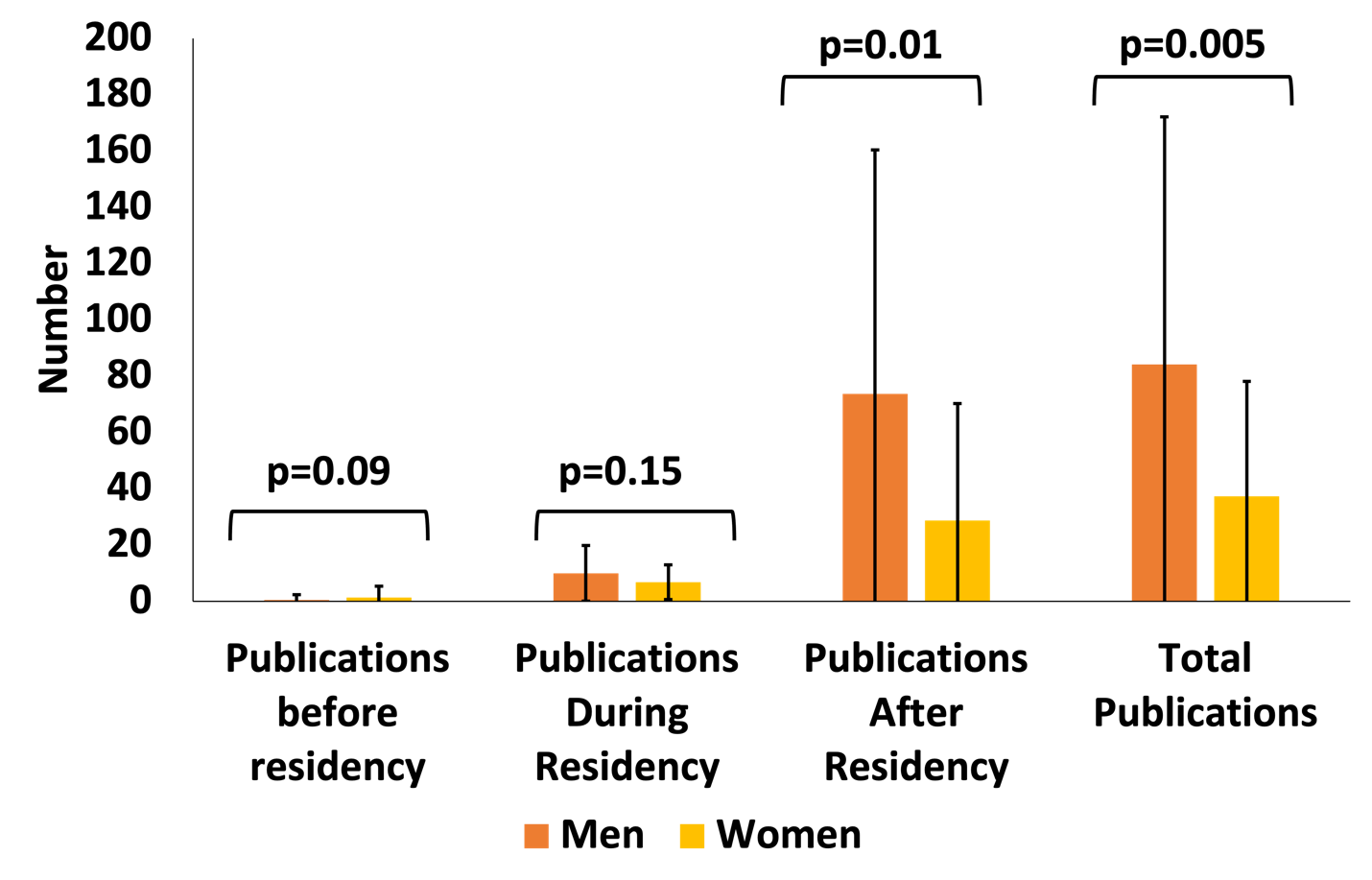


**Supplementary Data Figure 4**


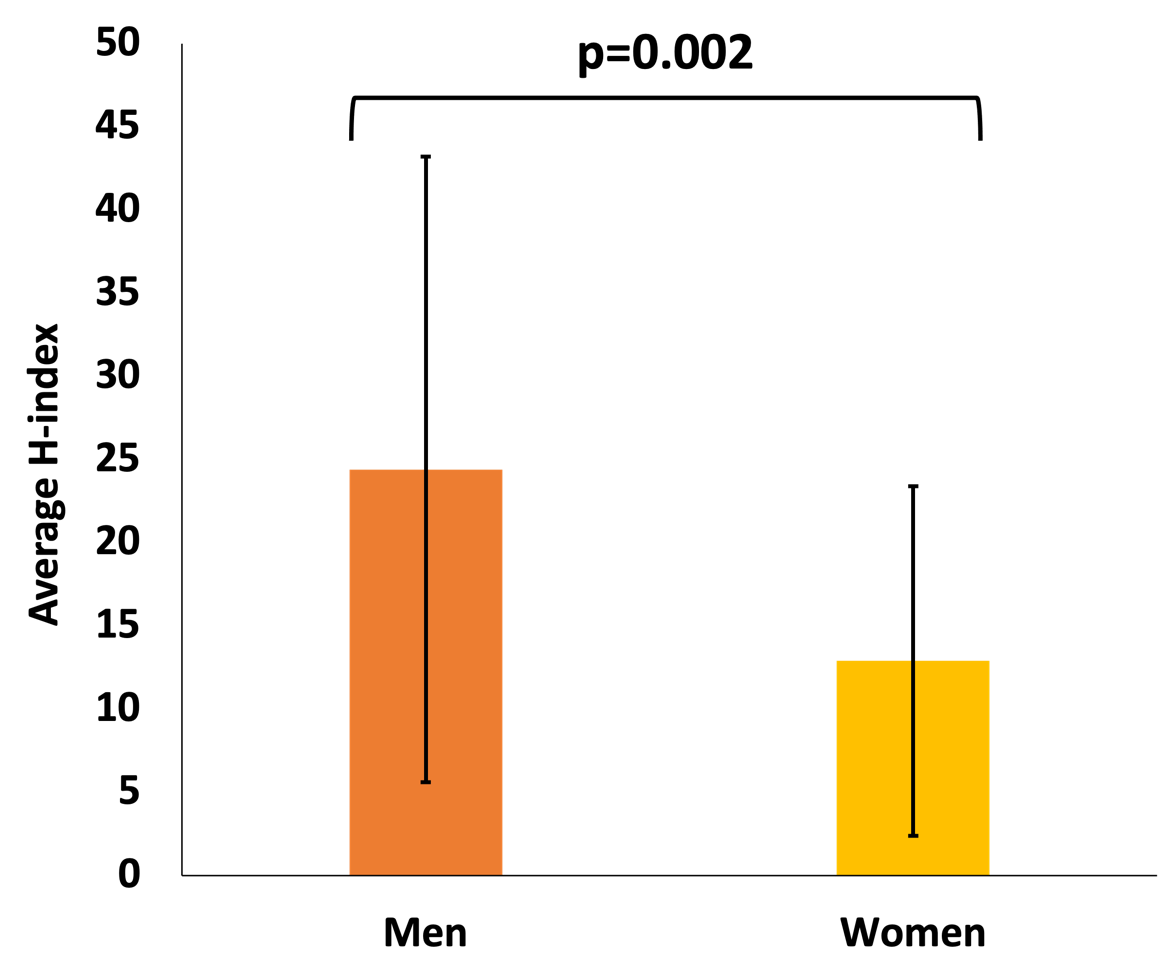


**Supplemental Data Figure 5**


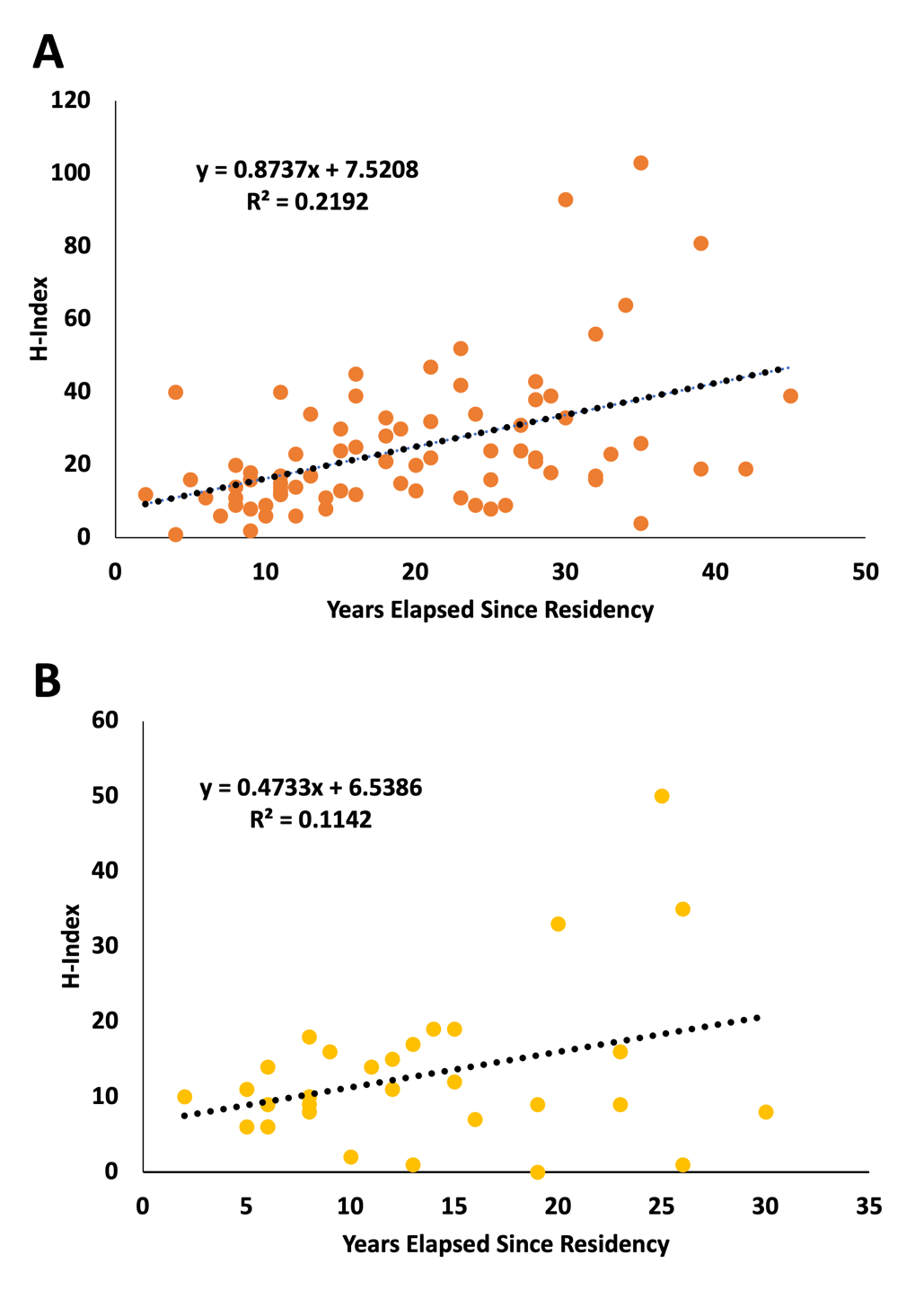


**Supplementary Data Figure Legends:**

**Supplementary Data Figure 1: Percentage of Women in the Pediatric Surgeon Training Pathway and Workforce as of 2023.** This data was compiled from reports published by the American Association of Medical Colleges (8) (27) (28) (29).

**Supplementary Data Figure 2: Our Study In Comparison to Pediatric Surgeon Workforce.** Our study analyzed approximately 10% of the pediatric surgeon workforce. As of 2023, 28% of pediatric surgeons were women [8]. In our study of 111 pediatric surgeons, 28% were women.

**Supplementary Data Figure 3: Publication Trend for Pediatric Surgeons by Gender.** P-values were calculated using a two-sided Student’s t-test.

**Supplementary Data Figure 4: H-Index for Pediatric Surgeons by Gender.** For the 111 pediatric surgeons we examined, women had significantly lower h-indexes in comparison to men (p=0.002).

**Supplemental Data Figure 5: H-Index vs. Years Elapsed Since Residency by Gender. A**) H-index versus years elapsed since residency for men; y=0.8737x+7.521, R^2^=0.2192, F=21.90, p<0.0001. **B**) H-index versus years elapsed since residency for women; y=0.4733x+6.5386, R^2^=0.1142, F=3.737, p=0.0630
